# Supplementary material for: HIV-Associated Insults Modulate ADAM10 and Its Regulator Sirtuin1 in an NMDA Receptor-Dependent Manner
Source: Cells. 2022 Sep 22;11(19):2962. doi: 10.3390/cells11192962 (PMC9564041; doi:10.3390/cells11192962)
Supplement: Supplementary file 1 [file cells-11-02962-s001.zip › cells-1855709-supplementary.pdf]

Supplementary data

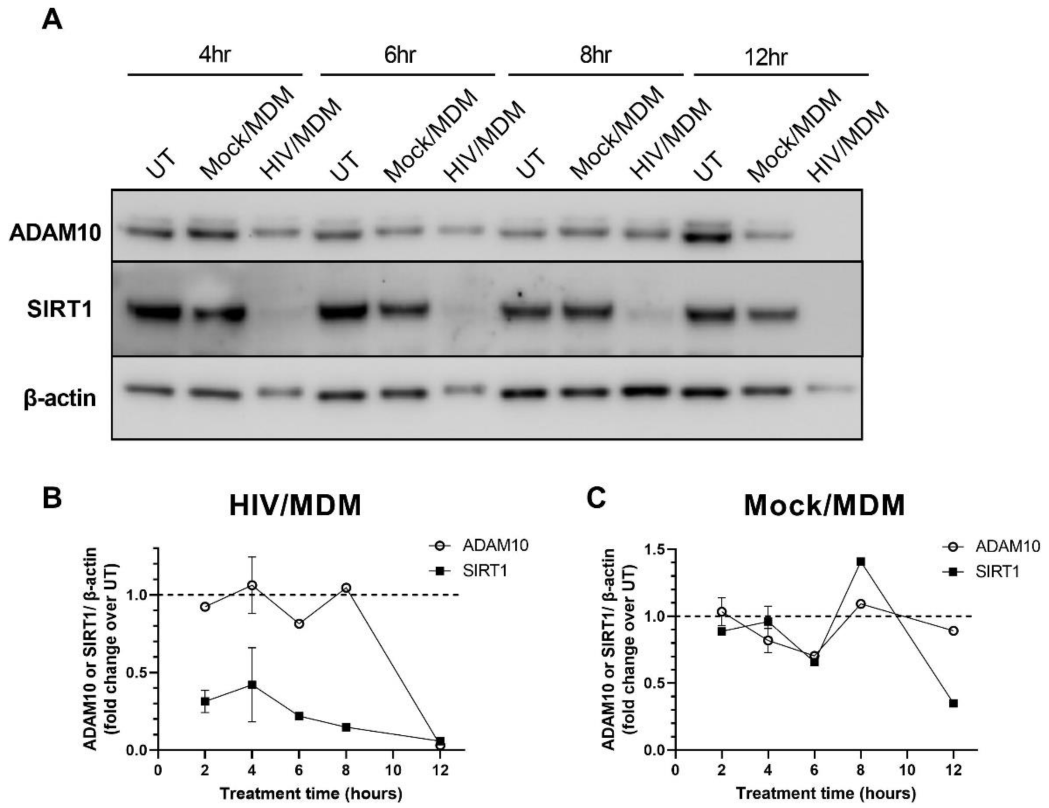

Figure S1. Mock and HIV/MDM time course. Primary rat neuronal cultures were treated with Mock and HIV/MDM. Cells were harvested 4, 6, 8 or 12 hours after treatment (**a**). SIRT1 decreases prior to ADAM10 as early as 2 hours in HIV/MDM-treated cells (**b**). ADAM10 and SIRT1 do not decrease in response to Mock/MDM supernatants prior to 12 hours after treatment (**c**).

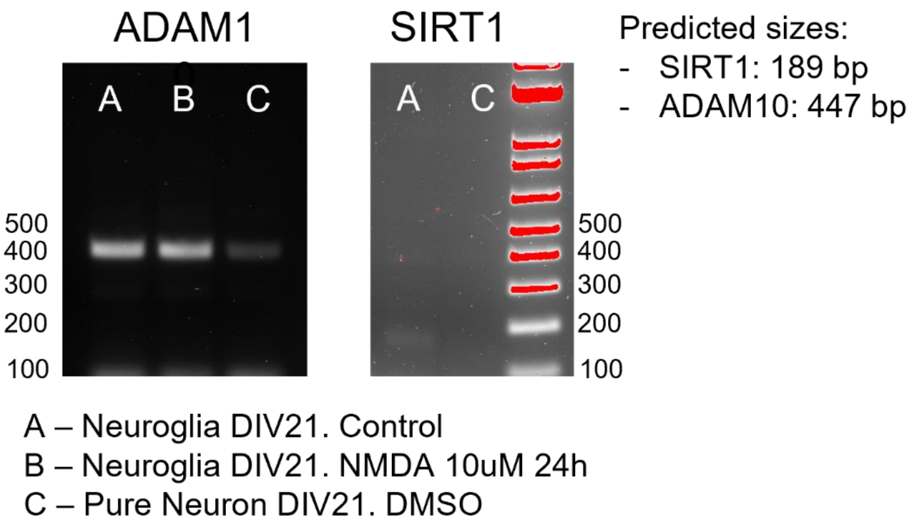

Figure S2. PCR for ADAM10 and SIRT1 from mRNA isolated and extracted from three different samples: A- untreated neuroglial cultures, B- neuroglial cultures treated with NMDA and C-pure neuronal cultures treated with DMSO vehicle.
